# Spontaneous pregnancy in a woman with diminished ovarian reserve following dietary supplementation with major royal jelly proteins: A case report

**DOI:** 10.1097/MD.0000000000049345

**Published:** 2026-06-19

**Authors:** Xiangyou Pi, Yufeng Yang, Qianqian Guo, Lirong Shen

**Affiliations:** a Institute of Life Sciences, School of Biological Science and Engineering, Fuzhou University, Fuzhou, Fujian Province, China; b Department of Social Medicine, School of Public Health, Zhejiang University, Hangzhou, Zhejiang Province, China; c Department of Food Science and Nutrition, School of Biosystems Engineering and Food Science, Zhejiang University, Hangzhou, Zhejiang Province, China.

**Keywords:** diminished ovarian reserve, major royal jelly proteins, royal jelly

## Abstract

**Rationale:**

Royal jelly (RJ), a natural secretion produced by the hypopharyngeal and mandibular glands of worker honeybees, is a nutrient-rich substance that contains proteins, lipids, vitamins, and bioactive compounds. Among its key components, major royal jelly proteins (MRJPs) constitute the predominant fraction and have been linked to various physiological benefits. Our previous studies on female mice have shown that MRJPs can increase uterine and ovarian indices, estradiol levels, and endometrial thickening. Overexpression of MRJPs in female *Drosophila* can increase estrogen levels and activate cell proliferation signaling pathways, thereby promoting body growth. Therefore, we speculate that MRJPs may be helpful for women. Here, we describe a woman with diminished ovarian reserve (DOR) who conceived naturally at week 4 after receiving oral MRJPs, an observation that warrants further investigation.

**Patient’s concerns:**

A 35-year-old female who was diagnosed with DOR through hormone testing before MRJPs administration. The clinical manifestations included dysmenorrhea and poor sleep. After taking the MRJPs, she conceived naturally.

**Diagnoses:**

The patient underwent hormonal testing at a specialized hospital and was diagnosed with DOR.

**Interventions:**

The patient started taking 1g lyophilized powder of MRJPs (provided by Hangzhou Aili Biotechnology Co., Ltd. using ultrafiltration-mediated enrichment to retain MRJPs with a molecular weight of 4.9 × 10^4^) orally in the morning and evening, respectively.

**Outcomes:**

The patient conceived naturally following 4 weeks of continuous MRJPs supplementation. During the administration of MRJPs, patients were monitored and asked about gastrointestinal and allergy-related adverse reactions. The results showed that no confirmed cases of gastrointestinal discomfort (including bloating, dull pain, cramps, nausea, or diarrhea) or allergic reactions (skin erythema, pruritus, or urticaria) occurred throughout the entire observation period.

**Lessons:**

Given the non-generalizable nature of this single-case report, a randomized double-blind clinical trial has been arranged. The mechanisms of MRJPs require further elucidation, and potential lifestyle confounders such as sleep quality and physical activity were not objectively monitored.

## 1. Introduction

Royal jelly (RJ) is secreted by the hypopharyngeal and mandibular glands of nurse worker bees and is rich in protein, which accounts for 9% to 18% of its content, along with sugars, small amounts of lipids, vitamins, salts, and free amino acids.^[1]^ Analyses of lyophilized protein powder extracted from fresh RJ showed that 82% to 90% (w/w) is constituted by major royal jelly proteins (MRJPs).^[1,2]^ The MRJPs powder with high purity was very stable at room temperature and was primarily composed of 9 soluble protein members exhibiting distinct amino acid compositions: MRJP1 (essential amino acid [EAA] content: 48%), MRJP2 (EAA content: 47%), MRJP3 (EAA content: 39.3%), MRJP4 (EAA content: 44.5%), MRJP5 (EAA content: 51.4%), MRJP6 (EAA content: 42%), MRJP7 (EAA content: 48.3%), MRJP8 (EAA content: 49.5%), and MRJP9 (EAA content: 47.3%).^[1,2]^ MRJP5 is rich in arginine and methionine; the main amino acids in MRJP1, MRJP2, and MRJP4 are leucine and valine; the main amino acids in MRJP3 are arginine and lysine; the main amino acids in MRJP6, MRJP7, and MRJP8 are leucine; and the main amino acid in MRJP9 is isoleucine.^[3]^ The composition of RJ varies according to the season and ecological conditions of the foraging site.^[4]^ Several studies have demonstrated that RJ can improve the pregnancy rate and litter size in ewes,^[5,6]^ enhance egg production in poultry,^[7]^ and increase the reproductive capacity of female *Drosophila*.^[8]^ Supplementation with MRJPs extends the lifespan of *Drosophila*, improves feeding efficiency and fertility in both sexes^[9]^ and increases serum estradiol (E2) levels in perimenopausal mice. Additionally, MRJPs elevate the uterine and ovarian indices and promote follicular development.^[10,11]^

Human infertility is defined as the inability to conceive successfully despite regular sexual intercourse for more than 1 year.^[12]^ Potential factors contributing to infertility in women of childbearing age include advanced childbearing status, endocrine disorders, ovarian insufficiency, uterine adnexal-related infections, and other unexplained causes. A diminished ovarian reserve (DOR) refers to a reduction in the number of recruitable follicles in the ovaries or a decline in oocyte quality, leading to decreased gamete production and reduced fertility. In China, due to delayed childbearing, the 2-child policy, and social environmental factors, the proportion of patients with DOR is increasing, accounting for 10% of infertile women.^[13]^ In recent years, the prevalence of infertility has continually increased from 12% in 2007 to 18% in 2020 in China. However, with advances in assisted reproductive technology, the success rate of infertility treatment has increased from 26% in 1990 to 40% to 60% in 2020. However, some patients do not have children for unknown reasons.^[13]^

RJ is marketed globally as a functional “Superfood” and cosmetic,^[14]^ with documented bioactivities (antimicrobial, antioxidant, anti-inflammatory, and immunomodulatory).^[15-18]^ However, no clinical evidence exists for MRJPs that improve natural pregnancy in women with infertility. Here, we present the first reported case of successful natural pregnancy and delivery in a woman with DOR, facilitated by MRJP supplementation.

## 2. Materials and methods

### 2.1. Hormonal assays

In accordance with the protocol described in the literature,^[19,20]^ hormonal assays were performed by Chain Medical Labs. Fasting blood samples were collected in the morning. For menstruating patients, venous blood (4 mL) was drawn on cycle day 3. Serum, obtained by centrifuging non-anticoagulated blood at 3000 rpm/min, was analyzed for levels of follicle-stimulating hormone (FSH), luteinizing hormone (LH), progesterone (PGN), E2, testosterone (TE), and prolactin (PRL) using an ADVIA Centaur CP automated chemiluminescence immunoassay analyzer with matched reagent kits. Anti-Müllerian Hormone (AMH) levels were measured using the matched direct chemiluminescence assay kit (SIEMENS Healthineers, 10998433). Supplementary File 1, Supplemental Digital Content 1 provides the patient’s medical records, which include the test results of these hormones as above.

### 2.2. Observation indices and efficacy evaluation criteria for hormones

The normal reference ranges were as follows:

FSH – follicular phase: 3.03 to 8.08 IU/L; ovulatory phase: 2.55 to 16.69 IU/L; luteal phase: 1.38 to 5.47 IU/L; postmenopausal: 26.72 to 133.41 IU/L.

LH – follicular phase: 1.80 to 11.78 IU/L; ovulatory phase: 7.59 to 89.08 IU/L; luteal phase: 0.56 to 14.0 IU/L; postmenopausal: 5.16 to 61.99 IU/L.

PGN – **f**ollicular phase: <0.95 nmol/L; ovulatory phase: 1.91 to 8.30 nmol/L; luteal phase: 3.82 to 50.56 nmol/L; postmenopausal: 0.00 to 0.64 nmol/L.

E2 – fFollicular phase: 77.1 to 921.2 pmol/L; ovulatory phase: 139.5 to 2381.8 pmol/L; luteal phase: 77.1 to 1145.0 pmol/L; postmenopausal: <102.8 pmol/L.

TE: 0.38 to 1.97 nmol/L.

PRL: 5.18 to 26.53 ng/mL.

AMH – aged 20 to 24 years: 1.52 to 9.95 ng/mL; 25 to 29 years: 1.20 to 9.05 ng/mL; 30 to 34 years: 0.711 to 7.59 ng/mL; 35 to 39 years: 0.405 to 6.96 ng/mL; 40 to 44 years: 0.059 to 4.44 ng/mL; 45 to 50 years: 0.01 to 1.79 ng/mL.

### 2.3. ThCG measurement

Serum total human chorionic gonadotropin (ThCG) levels were measured at the Hangzhou Obstetrics and Gynecology Hospital using a direct chemiluminescence immunoassay kit (SIEMENS Healthineers, 00643953) on an ADVIA Centaur CP analyzer. Briefly, the assay employed an acridinium ester-labeled polyclonal antibody and paramagnetic particle-coupled monoclonal antibody in a sandwich format. After incubation, magnetic separation and washing steps were performed, followed by the sequential addition of acid and base reagents to initiate the chemiluminescent reaction, which was then quantified by the analyzer. The normal reference range for ThCG is 0.0 to 10.0 IU/L. Supplementary File 1, Supplemental Digital Content 1 provides the patient’s medical records, which include the test results for ThCG.

### 2.4. IVF-ET procedure

In vitro fertilization-embryo transfer (IVF-ET) procedures were performed in accordance with established expert consensus guidelines.^[21,22]^ A GnRH agonist long protocol was used for ovarian stimulation, initiated in the mid-luteal phase of the preceding cycle. Gonadotropin stimulation was administered upon confirmation of down-regulation. Final oocyte maturation was triggered with hCG when the leading follicle reached 18 to 20 mm in diameter.

Oocytes were retrieved by transvaginal ultrasound-guided aspiration approximately 36 hours post-trigger. Semen samples were processed by density gradient centrifugation or swim-up. Fertilization was achieved either by conventional IVF co-incubation or by intracytoplasmic sperm injection in cases of severe male factor or prior fertilization failure. Resultant embryos were cultured under standard conditions for 3 to 6 days. Luteal phase support with PGN was administered following the procedure.

### 2.5. Ultrasonographic examination

Ultrasonographic examinations were performed at the Hangzhou Obstetrics and Gynecology Hospital. A systematic transabdominal scan was conducted with the patient in a supine position.

#### 2.5.1. Two-dimensional grayscale ultrasound

This initial assessment included evaluation of uterine position and morphology, fetal biometry (crown-rump length and nuchal translucency), a basic fetal anatomical survey (encompassing structures of the head, thorax, abdomen, and limbs), and assessment of placental location and amniotic fluid volume.

#### 2.5.2. Color doppler flow imaging (CDFI)

Color Doppler flow imaging was employed to evaluate fetal hemodynamics, including measurement of fetal heart rate and assessment of blood flow in the umbilical artery and ductus venosus.

#### 2.5.3. Three-/four-dimensional ultrasonography

When technically feasible, 4-dimensional ultrasonography was utilized to acquire dynamic volumetric datasets for enhanced evaluation of superficial fetal anatomy. Supplementary File 1, Supplemental Digital Content 1 provides the patient’s medical records, which include the ultrasound test results. Supplementary File 2, Supplemental Digital Content 2 provides the patient’s treatment timeline.

### 2.6. Preparation of MRJPs

Fresh RJ was dissolved in phosphate-buffered saline (PBS) and filtered through an ultrafiltration system. The system was fitted with 2 molecular weight cutoff (MWCO) membranes in sequence: first, a membrane with a 1 × 10^5^ MWCO to remove high-molecular-weight impurities, followed by a membrane with a 4.9 × 10^4^ MWCO to retain the MRJPs. The resulting protein concentrate was collected, subjected to vacuum freeze-drying to obtain a lyophilized powder, and stored at −80°C. Supplementary File 3, Supplemental Digital Content 3 provides the microbiological and various physicochemical indices for MRJPs (English Version). Supplementary File 4, Supplemental Digital Content 4 provides various antibiotic testing results for MRJPs (English Version). Supplementary File 5, Supplemental Digital Content 5 provides various hormone testing results for MRJPs (English Version). Supplementary File 6, Supplemental Digital Content 6 provides the microbiological and various physicochemical indices for MRJPs (Chinese Version). Supplementary File 7, Supplemental Digital Content 7 provides various antibiotic testing results for MRJPs (Chinese Version). Supplementary File 8, Supplemental Digital Content 8 provides various hormone testing results for MRJPs (Chinese Version).

## 3. Case presentation

A 35-year-old female patient was admitted to the hospital with a history of spontaneous abortion, accompanied by chronic dysmenorrhea and poor sleep quality. The patient led a strictly sedentary lifestyle, characterized by the absence of routine moderate-to-vigorous physical exercise. This low daily physical activity level is documented as it represents a potential modulating factor for female endocrine function and metabolic health^[23]^ (Supplementary Files 1, Supplemental Digital Content 1 and 2, Supplemental Digital Content 2 provide a detailed timeline of the patient’s medical history and prior consultations). After failed attempts at pregnancy using phytotherapy and assisted reproductive techniques, the patient sought MRJP-assisted dietary therapy for infertility. Previously, the patient’s husband was tested in May 2019, and his sperm quality test results were normal, thus ruling out the possibility that the patient’s husband was responsible for the inability to have children. Between March 2020 and June 2020, the patient first sought help from a traditional Chinese medicine practitioner and was treated with medically supervised consumption of botanicals, dextran tablets, and Peibao Jiaonang. During the treatment period, the patient underwent an ultrasound examination in April 2020, which revealed an endometrial thickness of 0.5 cm, a uterine size of 3.7 × 3 × 3.7 cm, and follicles measuring 1.5 × 1.4 × 1.3 cm. In May of the same year, ultrasound testing showed her endometrial thickness was 0.4 cm, her uterine size was 4.0 × 3.8 × 2.8 cm, and follicles were measuring 1.3 × 1.3 × 1.1 cm. The patient did not experience a pregnancy after natural conception. The patient then sought help from the fertility center of her local hospital, where various hormone levels were measured, producing the following results: FSH level of 6.02 IU/L (follicular phase: 3.03–8.08 IU/L), LH level of 1.97 IU/L (follicular phase: 1.8–11.78 IU/L), PGN level of 0.3 nmol/L (follicular phase: <0.95 nmol/L), E2 level of 62 pmol/L (follicular phase: 77.1–921.2 pmol/L), TE level of 0.79 nmol/L (reference range: 0.38–1.97 nmol/L), PRL level of 10.17 ng/mL (reference range: 5.18–26.33 ng/mL), and anti-Müllerian hormone level of 0.72 ng/mL (reference range:0.711–7.59 ng/mL). On the basis of these results, the patient was diagnosed with DOR. The patient then underwent in vitro IVF-ET as an assisted reproductive treatment recommended by a fertility specialist. After ovulation retrieval, the embryos were prepared for transfer in October 2020. However, ultrasound showed that the patient’s endometrial thickness during the transfer window was only 0.6 cm, which did not meet the requirements for transfer, and the transfer was abandoned.

**Figure 1.**
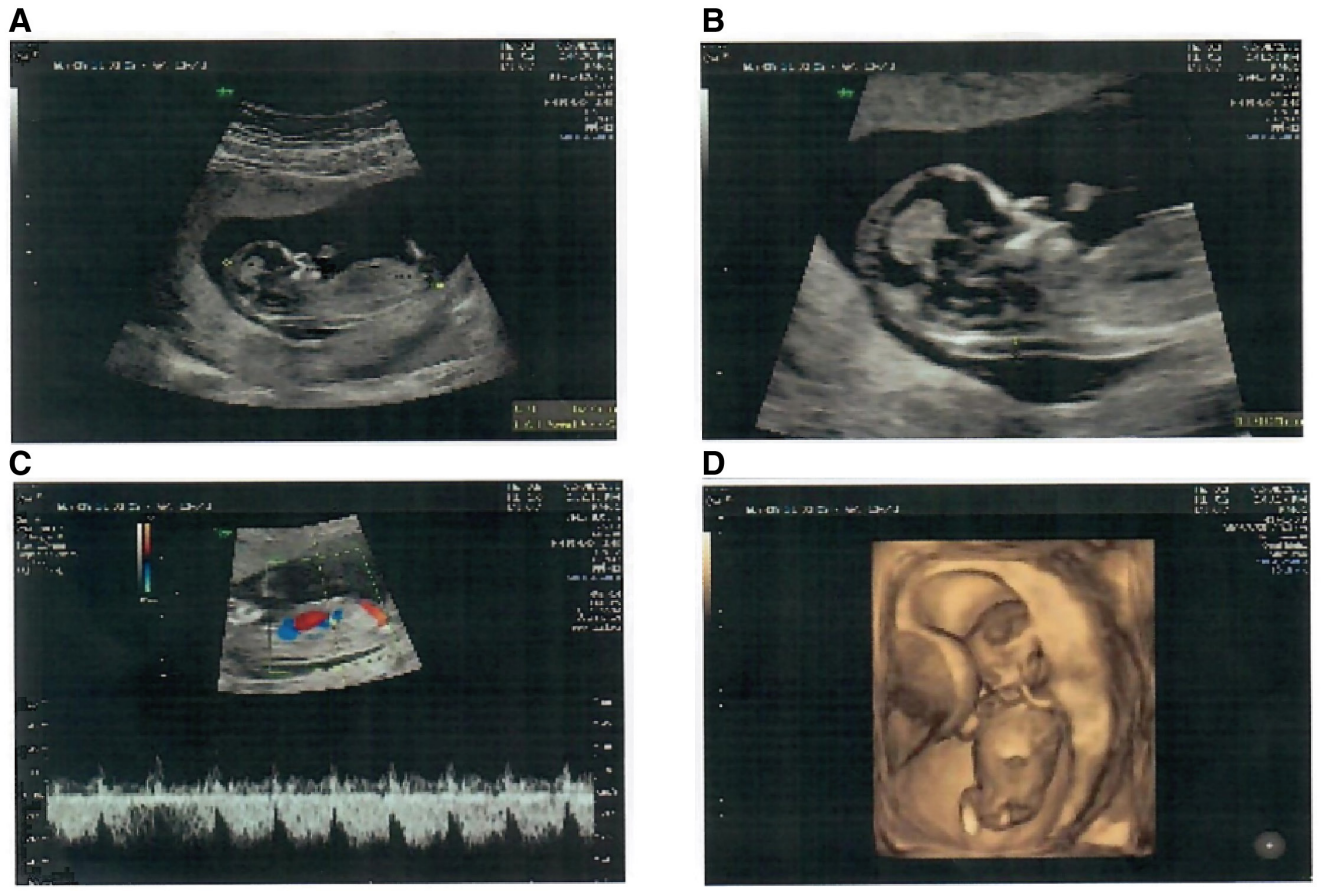
Sonographic findings of a confirmed intrauterine pregnancy. (A, B) Grayscale ultrasound images demonstrate an intrauterine fetus with a crown-rump length (CRL) of 64 mm, a nuchal translucency (NT) of 2.1 mm (within normal limits), and a visible nasal bone. Major anatomical structures, including the skull, midbrain, 4-chamber heart view, stomach, and all 4 limbs, are identified. The placenta is posterior, grade 0, with a thickness of 16 mm. Amniotic fluid distribution is normal. (C, D) Color Doppler (C) and 4-dimensional (D) images confirm fetal cardiac activity with a heart rate of 167 beats/min. The umbilical artery peak systolic velocity is approximately 40.7 cm/s, and venous duct flow shows no significant abnormality. CRL = crown-rump length, NT = nuchal translucency.

In November 2020, the patient started taking 1 g of MRJPs powder (invented technically by Zhejiang University and produced by Hangzhou Aili Biotechnology Co., Ltd.) twice daily in the morning and evening for fourth weeks (see Supplementary Files 3–8, Supplemental Digital Content 3–8 for product quality control reports on microbiology, purity, antibiotics, and hormones). The MRJPs with a molecular weight of 4.9 × 10^4^ was obtained by using ultrafiltration technology. On December 16, 2020, the patient’s total human chorionic gonadotropin level was measured, to be 2409.3 IU/L (normal range: 0.0–10.0 IU/L), indicating possible pregnancy. Pregnancy was subsequently confirmed using ultrasonography (Fig. 1), and a healthy boy was delivered in August 2021.

## 4. Discussion

This case report suggests that pregnancy in a woman with DOR occurred after the initiation of dietary supplementation with MRJPs. Although the patient had previously undergone phytotherapy and IVF-ET without significant improvement in 6 hormonal markers or endometrial ultrasound parameters during phytotherapy, pregnancy occurred spontaneously in the fourth week of the MRJPs intervention. Our previous research in female mice revealed that MRJPs may improve reproductive health in female mice through estrogen-like effects, receptor modulation, and antioxidant pathways.^[10,11]^ In naturally aged female mice (a model of human perimenopause), administration of high-dose MRJPs (250–500 mg/kg) for 7 weeks significantly increased serum E2 levels while reducing FSH and LH levels, and enhanced endometrial thickness.^[10,11]^ Additionally, medium-dose MRJPs (250 mg/kg) increased ovarian and uterine indices by 32.1% and 13.1%, respectively, and improved follicular development and morphology, promoting follicular maturation.^[10,11]^ In the puberty-onset model, immature ICR female mice gavaged with MRJPs for 45 days showed significantly elevated serum E2 levels, earlier onset of puberty (as indicated by vaginal opening), and accelerated follicular development (with a notable increase in the number of secondary and mature follicles).^[10,11]^ Mechanistically, MRJPs significantly upregulated the gene expression levels of estrogen receptors (ERs) and progesterone receptors (PRs) in ovarian tissues in both models, reflecting receptor-mediated regulation. Furthermore, MRJPs were found to reduce malondialdehyde levels and increase the activities of superoxide dismutase and glutathione peroxidase in ovarian tissues, indicating their regulatory role in antioxidant pathways.^[10,11]^ Perimenopause is characterized by declining ovarian function, reduced E2 secretion, and elevated pituitary gonadotropin (FSH and LH) levels, leading to a hypothalamic-pituitary-ovarian (HPO) imbalance. MRJPs supplementation appears to restore homeostasis of the HPO axis via the E2-mediated negative feedback inhibition of pituitary gonadotropin release.^[24]^ Mechanistically, MRJPs (250 mg/kg via gavage for 4 weeks)^[25]^ significantly enhanced the relative abundance of gut microbiota, including *Lactobacillus*, *Bifidobacterium*, and *Bacteroides*. These bacteria ferment dietary fiber to produce short-chain fatty acids such as acetate, propionate, and butyrate, which mediate systemic anti-inflammatory effects and modulate estrogen metabolism.^[26-28]^ Meanwhile, ectopic overexpression of MRJP1, MRJP2, MRJP3, and MRJP5 in *Drosophila* neurons markedly activates cell proliferation-associated pathways and leads to a significant increase in body size. Subsequent weighted gene co-expression network analysis and gene set enrichment analysis revealed that MRJP1, MRJP2, MRJP3, MRJP5, and MRJP7 converge on a shared set of target genes, including estrogen-responsive genes, to cooperatively activate proliferative signaling pathways, thereby promoting organismal growth.^[29]^ However, it is crucial to explicitly state that these findings from mouse and *Drosophila* models cannot be directly extrapolated to prove a causal mechanism in this single human case. While they provide a plausible biological hypothesis for the observed clinical outcome, the complex physiology of human infertility, particularly in DOR, involves multifaceted factors not fully captured in these animal models.

This case report describes a 35-year-old patient with secondary infertility and a history of recurrent miscarriage. The previous fertility treatments she had undergone were unsuccessful and may have been associated with certain side effects and physical and psychological distress. In contrast, MRJPs therapy demonstrated a favorable tolerance profile, causing minimal or no pain or side effects. Given that chronic psychological stress is a well-established risk factor for infertility,^[30]^ a better-tolerated treatment regimen may have been more conducive to the patient’s physical and mental well-being. It is plausible that the reduction in treatment-related stress contributed to a more optimal physiological state for conception, as research shows that even short-term interventions improving cognitive or psychological states can confer tangible physiological benefits.^[31]^ This could have created more favorable conditions for achieving a successful pregnancy. Meanwhile, when interpreting this outcome, the temporal proximity to the failed IVF-ET cycle (October 2020) must be considered a potential confounding factor. Ovarian stimulation during IVF can transiently perturb the HPO axis, and a “carry-over effect” on the subsequent cycle, though uncommon, is biologically plausible and cannot be entirely ruled out in this case.^[32]^ However, several contextual factors may argue against this being the sole explanation. The patient experienced a spontaneous menstrual cycle following the abandoned IVF attempt, which typically signifies a reset of the endocrine axis. Furthermore, exogenous gonadotropins used in IVF have a relatively short half-life, making a sustained, direct hormonal effect into the subsequent cycle less likely. Therefore, while the influence of the prior IVF cycle remains a consideration, conception may also be viewed as a discrete event within her ongoing fertility journey. It is plausible that the patient’s success was facilitated by a combination of factors, including the preparatory physiological changes from the IVF cycle and the concurrent MRJPs supplementation. This case highlights that natural conception shortly after an unsuccessful IVF cycle is a possibility, and patients might benefit from not forgoing this opportunity, potentially enhanced by supportive interventions.

This case report had several limitations. First, the patient’s sedentary lifestyle and low physical activity level, a recognized modulator of the endocrine profile, were not controlled for or quantitatively assessed beyond subjective reporting. This unmeasured variable could have interacted with the MRJPs supplementation or independently influenced the reproductive outcomes. Second, sleep disturbances are known to disrupt circadian rhythms and hormonal cascades, notably affecting the HPO axis.^[33]^ This dysregulation may lead to abnormalities in the secretion patterns and levels of FSH, LH, and E2, consequently contributing to menstrual cycle irregularities, ovulatory disorders, and luteal phase deficiency.^[34,35]^ Although sleep quality was not systematically monitored during the intervention period, it remained a potential confounding variable that should be considered when interpreting the observed outcomes. To address these issues in future studies, the use of modern objective monitoring tools, such as wearable technology, is highly recommended to provide precise, real-time data on sleep, physical activity, and physiological parameters, thereby effectively controlling for these confounders.^[36]^

This case raises fundamental questions for future research. First, which specific MRJPs components are bioavailable and mediate the fertility-enhancing effects? Second, to what extent do MRJPs exert their benefits through modulation of the gut-brain axis?

## 5. Conclusion

In summary, this case report presents an observation of natural pregnancy following MRJPs supplementation in an infertile woman. This observation warrants further investigation to define the potential role of MRJPs in the management of female infertility.

## Acknowledgments

We thank the patients and their families for their participation in the study. The authors declare that they have no conflicts of interest.

## Author contributions

**Conceptualization:** Yufeng Yang, Qianqian Guo, Lirong Shen.

**Data curation:** Qianqian Guo, Lirong Shen.

**Formal analysis:** Xiangyou Pi, Qianqian Guo.

**Supervision:** Lirong Shen.

**Writing – original draft:** Xiangyou Pi, Yufeng Yang.

**Writing – review & editing:** Xiangyou Pi, Yufeng Yang.
















