# Supplementary material for: Spontaneous pregnancy in a woman with diminished ovarian reserve following dietary supplementation with major royal jelly proteins: A case report
Source: Medicine (Baltimore). 2026 Jun 19;105(25):e49345. doi: 10.1097/MD.0000000000049345 (PMC13286341; doi:10.1097/MD.0000000000049345)
Supplement: Supplementary file 1 [file medi-105-e49345-s001.pdf]

杭州市中医院
门诊病历

姓名 [illegible]　性别：女　就诊卡号：[illegible]　血压：　mmHG　脉搏：　次/分

就诊日期：2020-03-23 10:00　就诊医生：马京　就诊科室：妇二科门诊（中医）　病人去向：离院

病人主诉：未避孕1年未孕

现病史：4-5/30天，LMP2.23，量多，血块-痛经-乳房胀痛
经艾灸治疗痛经好转，睡眠差，入睡困难，脚冷，胃口大小便正常，左头痛偶作。

体格检查：生命体征平稳，心肺腹无殊，58kg，168cm，舌淡苔薄白，脉弦

中医症象

病　　因：素体不足，运动少，睡眠迟

病　　机：患者肾精亏虚，脾胃虚弱，胞宫失养，故难以受孕

治　　则：健脾补肾助孕

方　　药：养巢方

辅助检查：(2019-05-09)，雌二醇(oE2) 128.14pg/ml，孕酮(PRGE) 28.05nmol/l
6.11超声：EM 0.7，右卵巢内黄体
1.23输卵管通液正常

既往史：否认药物食物过敏史，否认乙肝等传染病史，否认高血压、DM等内科疾病史，否认手术，外伤史

婚育史：0-0-1-0，药流

诊　　断：月经不规则

中医诊断：月经病类

处　　理：

草药方

注意事项：随诊。

医生签名：

扫描全能王
3亿人都在用的扫描App

杭州市中医院
门诊病历

姓名：　性别：女　就诊卡号 [illegible]　血压：　mmHG　脉搏：　次/分

就诊日期：2020-04-18 12:15　就诊医生 [illegible]　就诊科室：妇二科门诊（中医）　病人去向：离院

病人主诉：未避孕1年未孕

现 病 史：4-5/30天，LMP2.23，量多，血块-痛经-乳房胀痛
经艾灸治疗痛经好转。睡眠差，入睡困难，畏冷，胃口大小便正常，左头痛偶作。

体格检查：生命体征平稳，心肺听无殊，58kg，168cm，舌淡苔薄白，脉弦

中医症象

病　　因：素体不足，运动少，睡眠差

病　　机：患者肾精亏虚，脾胃虚弱，胞宫失养，故难以受孕

治　　则：健脾补肾助孕

方　　药：养巢方

辅助检查：(2019-05-09)，雌二醇(eE2) 128.14pg/ml，孕酮(PRGE) 28.05nmol/l
6.11超声：EM 0.7，右卵巢内黄体
1.23输卵管通液正常
4.18超声：EM 0.5cm，卵泡1.5*1.4*1.3CM，1.3*1.4*1.2
4.7超声：子宫3.7*3*3.7cm，卵泡1.4*1.3

既 往 史：否认药物食物过敏史，否认乙肝等传染病史，否认高血压、DM等内科疾病史，否认手术，外伤史

婚 育 史：0-0-1-0，药流

诊　　断：月经不规则

中医诊断：月经病

处　　理：子宫附件（周末-经腔内卵泡检测）
雌二醇/地屈孕酮片1毫克*28片　1片　口服　一次/日　1盒

注意事项：随诊。

医生签名：

扫描全能王
3亿人都在用的扫描App

杭州市中医院
门诊病历

姓名：[illegible]　性别：女　就诊卡号 [illegible]　血压：　mmHG　脉搏：　次/分

就诊日期：2020-04-28 13:54　就诊医生：马[illegible]　就诊科室：妇二科门诊（中医）　病人去向：离院

病人主诉： 未避孕1年未孕

现 病 史： 4-5/30天，LMP2.23，量多，血块-痛经-乳房胀痛
经艾灸治疗痛经好转，睡眠差，入睡困难，畏冷，胃口大小便正常，左头痛偶作，

体格检查： 生命体征平稳，心肺腹无殊，58kg，168cm，舌淡苔薄白，脉弦

中医描象

病　　因： 禀体不足，运动少，睡眠迟

病　　机： 患者肾精亏虚，脾胃虚弱，胞宫失养，故难以受孕

治　　则： 健脾补肾助孕

方　　药： 养巢方

辅助检查： (2019-05-09)，雌二醇(eE2) 128.14pg/ml，孕酮(PRGE) 28.05nmol/l
6.11超声：EM 0.7，右卵巢内黄体
1.23输卵管通液正常
4.18超声：EM 0.5cm，卵泡1.6*1.4*1.3CM，1.3*1.4*1.2
4.7超声：子宫3.7*3*3.7cm，卵泡1.4*1.3

既 往 史： 否认药物食物过敏史，否认乙肝等传染病史，否认高血压、DM等内科疾病史，否认手术，外伤史

婚 育 史： 0-0-1-0，药流

诊　　断： 月经不规则

中医诊断： 月经病`肾虚证

处　　理：

草药方

注意事项： 随诊，

医生签名：

扫描全能王
3亿人都在用的扫描App

杭州市中医院
门诊病历

姓名： 性别：女 就诊卡号： 血压： mmHG 脉搏： 次/分

就诊日期：2020-05-12 17:03 就诊医生：马[illegible] 就诊科室：妇二科门诊（中医） 病人去向：离院

病人主诉：未避孕1年未孕

现病史：4-5/30天，LMP2.23，量多，血块-痛经-乳房胀痛，经艾灸治疗痛经好转，lmp5.6，量中等，时胀发，入睡困难，畏冷，胃口大小便正常，左头痛偶作。

体格检查：生命体征平稳，心肺腹无殊，58kg，168cm，舌淡苔薄白，脉弦

中医症象

病　　因：素体不足，运动少，睡眠迟

病　　机：患者肾精亏虚，脾胃虚弱，胞宫失养，故难以受孕

治　　则：健脾补肾助孕

方　　药：养巢方

辅助检查：(2019-05-09)，雌二醇(eE2) 128.14pg/ml，孕酮(PRGE) 28.05nmol/l
6.11超声：EM 0.7，右卵巢内黄体
1.23输卵管通液正常
4.18超声：EM 0.5cm，卵泡1.5*1.4*1.3CM，1.3*1.4*1.2
4.7超声：子宫3.7*3*3.7cm，卵泡1.4*1.3

既往史：否认药物食物过敏史，否认乙肝等传染病史，否认高血压、DM等内科疾病史，否认手术，外伤史

婚育史：0-0-1-0，药流

诊　　断：月经不规则

中医诊断：月经病`肾虚证

处　　理：子宫附件（经腔内卵泡监测）
胚宝胶囊0.3克*36粒　3粒　口服(饭前)三次/日　2盒
体药方

注意事项：随诊。

医生签名：[illegible]

扫描全能王
3亿人都在用的扫描App

杭州市中医院
门诊病历

姓名：[illegible]　　性别：女　　就诊卡号：[illegible]　　血压：　　mmHG　脉搏：　　次/分

就诊日期：2020-05-19 16:17　　就诊医生：[illegible]　　就诊科室：妇二科门诊（中医）　　病人去向：离院

病人主诉：未避孕1年未孕

现 病 史：4-5/30天，LMP2.23，量多，血块-痛经-乳房胀痛，经艾灸治疗痛经好转，lmp5.6，量中等，睡眠差，入睡困难，畏冷，胃口大小便正常，左头痛偶作。

体格检查：生命体征平稳，心肺腹无殊，68kg，168cm，舌淡苔薄白，脉弦

中医症象

病　　因：素体不足，运动少，睡眠迟

病　　机：患者肾精亏虚，脾胃虚弱，胞宫失养，故难以受孕

治　　则：健脾补肾助孕

方　　药：养巢方

辅助检查：(2019-05-09)，雌二醇(cE2) 128.14pg/ml，孕酮(PRGE) 28.05nmol/l
6.11超声：EM 0.7，右卵巢内黄体
1.23输卵管通液正常
4.18超声：EN 0.5cm，卵泡1.5*1.4*1.3CM，1.3*1.4*1.2
4.7超声：子宫3.7*3*3.7cm，卵泡1.4*1.3
5.19超声：子宫4.0*3.8*2.8，EM 0.4cm，卵泡1.3*1.3*1.1

既 往 史：否认药物食物过敏史，否认乙肝等传染病史，否认高血压、DM等内科疾病史，否认手术，外伤史

婚 育 史：0-0-1-0，药流

诊　　断：月经不规则

中医诊断：月经病-肾虚证

处　　理：子宫附件（周末-经腔内卵泡检测）
[illegible]胶囊0.3克*36粒　　3粒　　口服(餐前)三次/日　　2盒
草药方

注意事项：随诊。

医生签名：[illegible]

扫描全能王
3亿人都在用的扫描App

杭州市中医院
门诊病历

姓名： 性别：女 就诊卡号： 血压： mmHG 脉搏： 次/分

就诊日期：2020-06-15 10:54 就诊医生：马[illegible] 就诊科室：妇二科门诊（中医） 病人去向：离院

病人主诉：未避孕1年未孕

现 病 史：4-5/30天，LMP2.23，量多，血块+痛经+乳房胀痛，经艾灸治疗痛经好转，Lmp5.6，LMP6.10，量中等，睡眠差，入睡困难，脚冷，胃口大小便正常，左头痛偶作。

体格检查：生命体征平稳，心肺腹无殊，58kg，168cm，舌淡苔薄白，脉弦

中医症象

病　　因：素体不足，运动少，睡眠迟

病　　机：患者肾精亏虚，脾胃虚弱，胞宫失养，故难以受孕

治　　则：健脾补肾助孕

方　　药：养巢方

辅助检查：(2019-05-09)，雌二醇(eE2) 128.14pg/ml，孕酮(PRGE) 28.05nmol/l
5.19超声：子宫4.0*3.8*2.8，EM 0.4cm，卵泡1.3*1.3*1.1

既 往 史：否认药物食物过敏史，否认乙肝等传染病史，否认高血压、DM等内科疾病史，否认手术，外伤史

婚 育 史：0-0-1-0，药流

诊　　断：月经不规则

中医诊断：月经病˙肾虚证˙血瘀证

处　　理：

胚宝胶囊0.3克*36粒 3粒 口服(餐前)二次/日 2盒

草药方

注意事项：随诊。

医生签名：[illegible]

扫描全能王
3亿人都在用的扫描App

杭州市中医院

精液及精子质量分析报告单

姓名	年龄: 38	门诊号:
编号:	环境温度: 36℃	日期: 2019-06-10
无精精人数:	存放时间(min): 45	液化时间(min, 正常<60): 45
颜色: 灰白	气味: 正常	粘稠度: -
PH值(正常≥7.2): 7.9	稀释比: 不稀释	轨迹描述时间: 1.00
精液量(正常≥1.5ml): 2.2	红细胞(个):	白细胞(个):

精子类别[V=VCL, S=STR]	总数[百万]	密度[百万/ml](正常)	百分比[%] (正常)
全部精子(计数: 333 个)	166	75.64 (≥15)	
活动精子	134.63	61.15	80.85 (≥40)
前进精子[2级+3级]	122.80	55.82	73.80 (≥32)
快速精子[V>=35]	84.83	38.56	50.98
中速精子[20<=V<35]	31.42	14.28	18.88
低速精子[V<20]	50.16	22.80	30.14
静止精子-0级	31.88	14.49	19.16
原地活动-1级(VSL<10)	11.73	5.33	7.05
慢速前进-2级(VSL>10)	90.46	41.12	54.36
快速前进-3级(VSL>30)	32.34	14.70	19.43
直线精子	0.00	0.00	0.00
优质精子(V>=35, S>=80)	21.56	9.80	12.96

活动精子运动特性:

曲线速度VCL(μm/s):	39.16	线性度(LIN):	41.67
路径速度VAP(μm/s):	26.17	摆动性(WOB):	66.83
直线速度VSL(μm/s):	16.32	前向性(STR):	62.35
平均移动角度(MAD):	17.92	头摆幅度(ALH):	0.86
鞭打频率(BCF):	5.31		

精子运动速度分布(um/s): 数量(百万)和百分比(%)

速度(um/s)	曲线(百万)	百分比(%)	路径(百万)	百分比(%)	直线(百万)	百分比(%)
0-10	19.92	12.00	[illegible].82	27.00	78.02	47.00
10-20	29.88	18.00	29.88	18.00	31.54	19.00
20-30	21.58	13.00	19.92	12.00	23.24	14.00
30-40	16.60	10.00	21.58	13.00	11.62	7.00
40-50	16.60	10.00	19.92	12.00	9.96	6.00
50-60	14.94	9.00	16.60	10.00	3.32	2.00
60-70	19.92	12.00	4.98	3.00	1.66	1.00
70-..	26.56	16.00	8.30	5.00	6.64	4.00

备注: 审核医师: 马[illegible] 检验医师: [illegible]

只作临床参考，不作证明材料

扫描全能王
3亿人都在用的扫描App

杭州千麦医学检验所有限公司检验报告单

Chain Medical Labs

姓名：[redacted]	条 形 码：[redacted]	门诊/住院号：[redacted]	送检单位：浙江中医药大学杭州滨江中医门诊部
性别：女	医院条码：/	科室/病区：/	临床诊断：/ 标本性状：外观正常
年龄：34 岁	标本类型：血清	床号： /	送检医生：印姣君 采集日期：2020-07-13 00:00

序号	简 称	检 验 项 目	结 果	提示	参考区间	单位
*1	FSH	卵泡刺激素	6.02		卵泡期：3.03-8.08<br>排卵期：2.55-16.69<br>黄体期：1.38-5.47<br>绝经后：26.72-133.41	IU/L
*2	LH	促黄体生成素	1.97		卵泡期：1.80-11.78<br>排卵期：7.59-89.08<br>黄体期：0.56-14.00<br>绝经后：5.16-61.99	IU/L
*3	PGN	孕酮	0.30		卵泡期：<0.95<br>排卵期：1.91-8.30<br>黄体期：3.82-50.56<br>绝经后：0.00-0.64	nmol/L
*4	E2	雌二醇	62.0		卵泡期：77.1-921.2<br>排卵期：139.5-2381.8<br>黄[illegible]：77.1[illegible]145.0<br>绝[illegible]后：<102.8	pmol/L
*5	TE	睾酮	0.79		[illegible]38-1.97	nmol/L
*6	PRL	泌乳素	10.17		[illegible].18-26.53	ng/ml
	（以下为空白）					

备注：/

带“*”[illegible]表示通过[illegible]

千麦医检 Chain Medical Labs CMLabs

接收日期：2020-07-13 20:04 报告日期：2020-07-14 00:59 检验者：[illegible] 审核者：[illegible]

扫描全能王
3亿人都在用的扫描App

CMLabs 千麦医检

杭州千麦医学检验所有限公司检验报告单

Chain Medical Labs

姓名: [redacted]	[redacted]形 码 [redacted]	门诊/住院号 [redacted]	送检单位: 浙江大学医学院附属邵逸夫医院（生殖中心）
性别: [redacted]	医院条码: 57[redacted]	科室/病区: 妇[redacted]	临床诊断:
			标本性状: 外观正常
年龄: 34 岁	标本类型: 血清	床号:	送检医生: 匡琳
			采集日期: 2020-07-06 13:25

序号	简 称	检 验 项 目	结 果	提示	参考区间	单位
1	AMH_R	抗缪勒氏管激素	0.72		20-24周岁：1.52-9.95<br>25-29周岁：1.20-9.05<br>30-34周岁：0.711-7.59<br>35-39周岁：0.405-6.96<br>40-44周岁：0.059-4.44<br>45-50周岁:0.01-1.79	ng/ml

（以下为空白）

扫描全能王
3亿人都在用的扫描App

杭州市妇产科医院(杭州市妇幼保健院)
杭州市第一人民医院钱江新城院区
疾病诊疗证明书

NO

健康号1[illegible]名[illegible]性别女，年龄[illegible]岁，曾在本院11F产科一区(钱江)诊治，入院日期2021-08-11，出院日期2021-08-16，兹将诊断及诊疗意见开列如后以资参考。

诊断：孕2产1孕39周ROA顺产活婴，结缔组织病，高龄初产

诊疗意见： 患者于我院 剖宫产分娩一活婴，特此证明

杭州市妇产科医院
出院医疗证明专用章

医师：________(签名及工号章)

2021-08-16

(此证明经盖章有效)

病案归档号：2[illegible]

扫描全能王
3亿人都在用的扫描App

杭州市妇产科医院(杭州市妇幼保健院)
杭州市第一人民医院钱江新城院区
出院记录

健康号:1[redacted]　姓名[redacted]　性别:女　科别:产科病区(钱江)　床号　病案归档号

姓名 [redacted]　性别:女　出生年月:1985-12-31　主诊医师:余春风

入院日期:2021-08-11　入院诊断：孕2产0孕38+6周ROA待产，结缔组织病，巨大儿？，高龄初产

出院日期:2021-08-16　出院诊断:孕2产1孕39周ROA难产活婴，结缔组织病，高龄初产

住院天数:5　疗效评价:治愈

入院情况:

1 [redacted] 女，35岁，0-0-1-0。2. 停经38+6周,入院待产。

住院诊治经过(包括重要发现和结论、接受手术和操作、药物和其它治疗):

入院后完善相关检查，于2021-08-12因“巨大儿？”行子宫下段剖宫产术，娩一活婴，手术过程顺利，术后恢复可，今予出院。

病理报告： 无

手术/操作： 子宫下段剖宫产术 （日期：2021-08-13 ） 植入物：无

住院主要使用药物： 头孢美唑、缩宫素、益母草颗粒等

药物不良反应： 无

出院情况：无明显不适主诉，一般情况可，手术部位无明显血肿，无红肿渗出。疗效:治愈

出院去向：回家

婴儿去向：√母婴同时出院 □转NICU □其它

出院带药： 益母草颗粒(无蔗糖)(5克*24袋)*1盒:1袋 冲服 1日2次

出院指导： 1.注意休息、饮食。2.产后2月内禁性生活及盆浴。3.产后42天母婴随访：周一至周五挂产后门诊。4.哺乳期禁服避孕药。5.建议休难产假，严格避孕2年。6.如有阴道出血量多、腹痛等不适及时就诊。7.风湿免疫科随诊。

复诊： 口不需要

口需要 随访方式：门诊 复诊时间： 产后42天 随访周期：产后42天

不适随诊： 是 咨询电话： 0571-56005111 地点： 门急诊

温馨提醒：

出院患者办理复印病历须在患者出院7个工作日后，周一至周五（上午8:00-12:00，下午13:30-17:00）住院部9楼病案统计室办理。患者本人办理复印病历须携带患者本人身份证原件，代理人则须提供患者及代理人双方身份证原件。

扫描全能王
3亿人都在用的扫描App

杭州市妇产科医院(杭州市妇幼保健院)
杭州市第一人民医院　钱江新城院区
新生儿母婴同室出院记录单

健康号　姓名　之婴儿 性别:男 科别:产科病区(钱江) 床号:　病案归档号

母亲姓名、　　　　　出生日期：2021/8/12 13:05:00

出院日期：2021-08-16

出院诊断：足月新生儿

治疗经过：母婴同室新生儿常规照护

出院情况：出院时体重：3480 克　　经皮测胆红素：16.9mg/dl

出院指导（医嘱）：

1、建议无特殊情况下纯母乳喂养至少6个月，遇到问题，建议母乳喂养门诊。

2、脐残端消毒1～2次/日，直至脐脱落、干燥。若脐部红肿、脓性分泌物、活动性出血，建议就诊。

3、注意黄疸程度和消退时间。

√目前经皮测胆红素值已（临近）超过正常范围，建议每天监测经皮测胆红素值。

4、出院后可前往所在的社区医疗机构建卡及随访。本院门诊五楼设新生儿科门诊，日间工作时间可提供新生儿非急诊的常见病、多发病诊治及经皮测胆红素测定；本院门诊六楼设保健门诊（周一至周六开放）提供保健咨询、生后42天健康检查等服务。

5、本院不设新生儿急诊、儿科门急诊，如发现少吃、少哭、少动、发热、呕吐、腹胀、腹泻、紫绀、惊厥、嗜睡及其他急诊异常请及时前往具有新生儿（儿科）急诊医疗机构就诊。

6、出院带药：√聚维酮碘溶液 5% 100ml /用法：少许 外用

√茵栀黄口服液1盒/用法：5毫升 口服 一天二次

√维生素AD滴剂1盒/用法：1粒 口服 一天一次（出生二周开始）

7、患儿今日晨测TCB 16.9mg/dL，已达光疗指征，需转新生儿科住院进一步治疗，告知家属若患儿黄疸继续加重，可导致胆红素脑病的发生，出现呕吐、尖叫、抽搐等新的症状，甚至遗留神经系统后遗症，家属表示知情理解，但仍拒绝住院治疗，签字为证：[redacted] √

医生签字：曾广平 曾广平　　时间：21年 8月16 日 10时 15 分

监护人意见：医生/护士已经与我就出院计划给予指导；我已收到书面出院计划和指导，了解以上所告知内容。医生已经告知我出院后可根据我自己的意愿，前往我所在的社区医疗机构复诊。

监护人签字：[redacted]　　时间：2021年 8月16日 10时20分

扫描全能王
3亿人都在用的扫描App

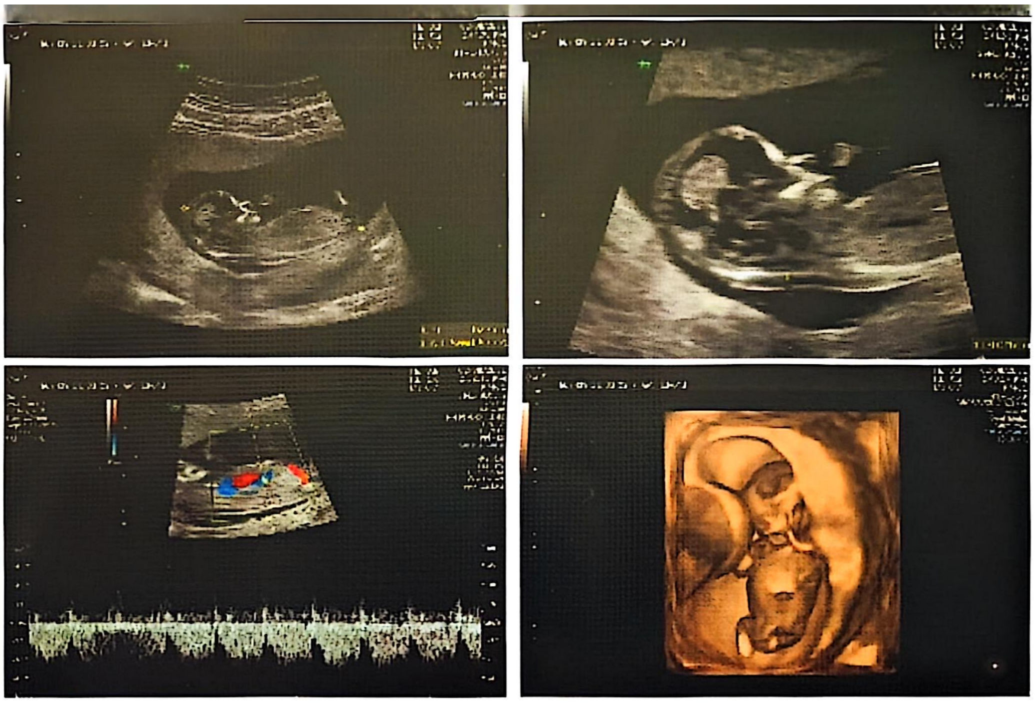

超声描述：

子宫前位，体积增大，宫壁回声均匀，宫腔内可见初具人形胎儿，可见胎儿肢芽，头臀径：64mm，胎儿颈项部透明层厚度（NT）：2.1mm，鼻骨可见。

胎儿颅脑及脑中线：可见；胎儿四腔心切面可显示。

胃泡：可见；膀胱：显示困难。

双上肢：可见；　　双下肢：可见。　　其他胎儿情况：无

胎盘附着于子宫后壁，成熟度：0级，脐带正常插入胎盘；胎盘厚度约：16mm。

羊水分布均匀，羊水最大前后径：37mm。

彩色多普勒显像（CDFI）：可见胎心搏动信号，脐动脉收缩期峰值血流速度约：40.7cm/s，胎儿心率：167次/分。静脉导管血流未见明显异常。

孕妇情况：双侧附件区未见明显异常回声。

备注：

1、本次检查属于产前常规超声检查，因胎龄小，胎儿颅内结构、颜面部、心脏、肾脏、脊柱等结构显示不清，请知情并理解。

2、受胎儿及母体因素影响，孕期超声检查有其局限性，检出率和准确率也不可能达到100%，应进行必要的复查。

3、孕妇已知情并签署了知情同意书。

超声诊断（仅供临床参考）：

宫内妊娠 单活胎

胎儿颈项部透明层厚度（NT）在正常值范围内（正常值<2.5mm）

根据胎儿生物学测量，估计孕龄约为12周+6天

扫描全能王
3亿人都在用的扫描App

HD 5G 10:48

门诊检验报告详情

姓名：**仟　　　　性别：

日期：2020-12-16　　　　年龄：

检查项目	结果	参考范围	单位
总人绒毛膜促性腺激素(ThCG)	2409.3	0.0-10.0	IU/L

数据来源于医院，本查询信息仅供参考
请以医院纸质报告单为准

扫描全能王
3亿人都在用的扫描App
