# Supplementary material for: Spontaneous pregnancy in a woman with diminished ovarian reserve following dietary supplementation with major royal jelly proteins: A case report
Source: Medicine (Baltimore). 2026 Jun 19;105(25):e49345. doi: 10.1097/MD.0000000000049345 (PMC13286341; doi:10.1097/MD.0000000000049345)
Supplement: Supplementary file 2 [file medi-105-e49345-s002.pptx]

## Slide 1
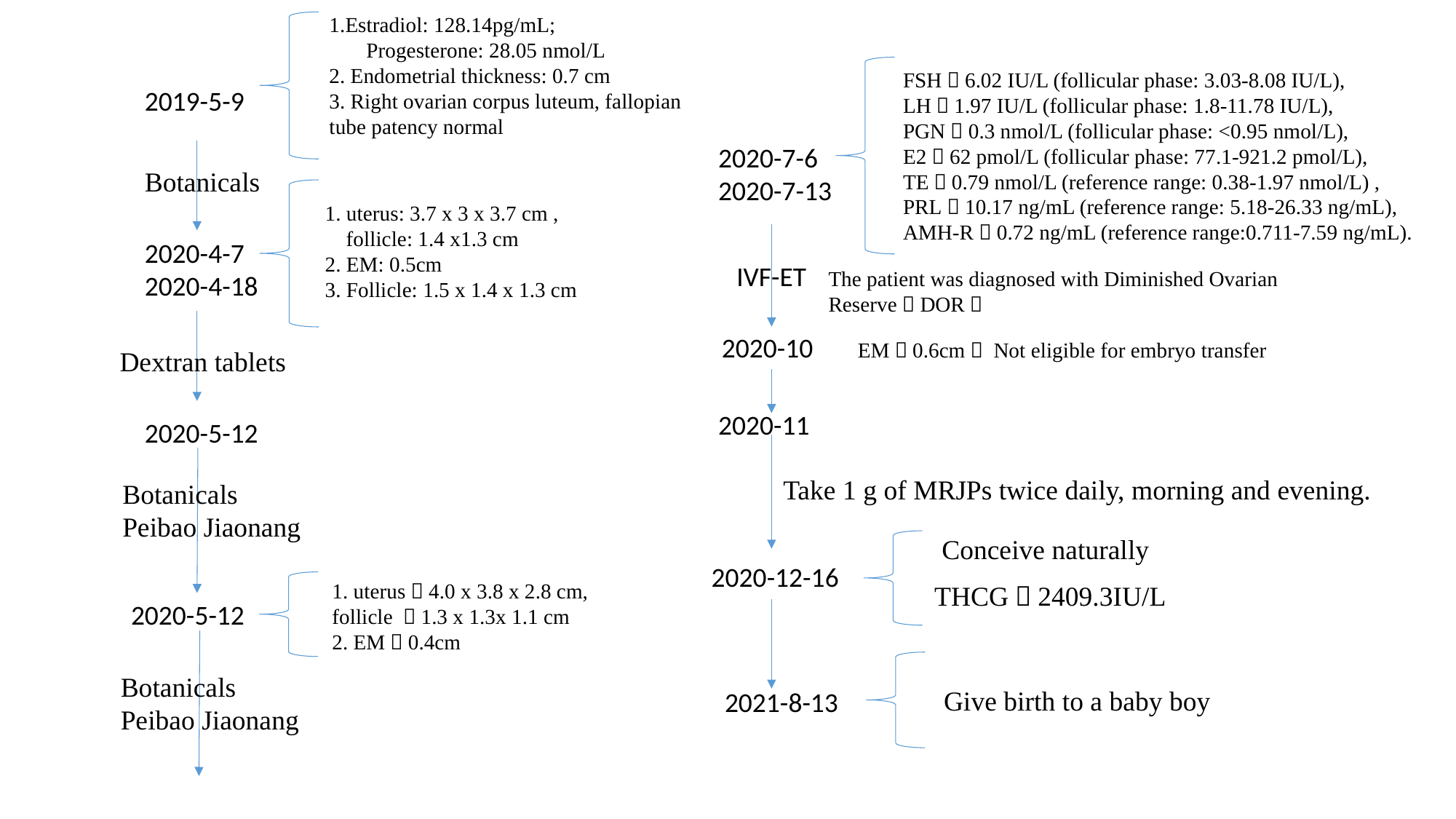

1.Estradiol: 128.14pg/mL;
 Progesterone: 28.05 nmol/L
2. Endometrial thickness: 0.7 cm
3. Right ovarian corpus luteum, fallopian tube patency normal
FSH：6.02 IU/L (follicular phase: 3.03-8.08 IU/L),
LH：1.97 IU/L (follicular phase: 1.8-11.78 IU/L),
PGN：0.3 nmol/L (follicular phase: <0.95 nmol/L),
E2：62 pmol/L (follicular phase: 77.1-921.2 pmol/L),
TE：0.79 nmol/L (reference range: 0.38-1.97 nmol/L) ,
PRL：10.17 ng/mL (reference range: 5.18-26.33 ng/mL),
AMH-R：0.72 ng/mL (reference range:0.711-7.59 ng/mL).
2019-5-9
2020-7-6
2020-7-13
Botanicals
1. uterus: 3.7 x 3 x 3.7 cm ,
 follicle: 1.4 x1.3 cm
2. EM: 0.5cm
3. Follicle: 1.5 x 1.4 x 1.3 cm
2020-4-7
2020-4-18
IVF-ET
The patient was diagnosed with Diminished Ovarian Reserve（DOR）
2020-10
EM：0.6cm， Not eligible for embryo transfer
Dextran tablets
2020-11
2020-5-12
Take 1 g of MRJPs twice daily, morning and evening.
Botanicals
Peibao Jiaonang
Conceive naturally
2020-12-16
1. uterus：4.0 x 3.8 x 2.8 cm,
follicle ：1.3 x 1.3x 1.1 cm
2. EM：0.4cm
THCG：2409.3IU/L
2020-5-12
Botanicals
Peibao Jiaonang
Give birth to a baby boy
2021-8-13
