# Supplementary material for: Spontaneous pregnancy in a woman with diminished ovarian reserve following dietary supplementation with major royal jelly proteins: A case report
Source: Medicine (Baltimore). 2026 Jun 19;105(25):e49345. doi: 10.1097/MD.0000000000049345 (PMC13286341; doi:10.1097/MD.0000000000049345)
Supplement: Supplementary file 3 [file medi-105-e49345-s003.pdf]

Testing Report QDF22-020361-03 Report date: May 05, 2022

Description of tested sample :

Sample Number	SGS sample ID.	Description
1	QDF22-020361.002	Bagged sample

Microorganism testing
Testing results

Testing items	Units	Testing methods	Testing results
Escherichia cola flora	CFU/g	GB 4789.3-2016 Method 2	<10
Escherichia cola flora	CFU/g	GB 4789.3-2016 Method 2	<10
Escherichia cola flora	CFU/g	GB 4789.3-2016 Method 2	<10
Escherichia cola flora	CFU/g	GB 4789.3-2016 Method 2	<10
*Escherichia col*a flora	CFU/g	GB 4789.3-2016 Method 2	<10
Salmonella	/25g	GB 4789.4-2016	ND
Salmonella	/25g	GB 4789.4-2016	ND
Salmonella	/25g	GB 4789.4-2016	ND
Salmonella	/25g	GB 4789.4-2016	ND
Salmonella	/25g	GB 4789.4-2016	ND
Total number of bacterial colonies	CFU/g	GB 4789.2-2016	<10
Total number of bacterial colonies	CFU/g	GB 4789.2-2016	<10
Total number of bacterial colonies	CFU/g	GB 4789.2-2016	<10
Total number of bacterial colonies	CFU/g	GB 4789.2-2016	<10
Total number of bacterial colonies	CFU/g	GB 4789.2-2016	<10

Microorganism testing
Testing results

Testing items	Units	Testing methods	Testing results
Mucedine	CFU/g	GB 4789.3-2016 Method 1	<10
Yeast	CFU/g	GB 4789.3-2016 Method 1	<10

SGS-CSTG Standards & Testing
Services (Qingdao) Co., Ltd.

Testing Report QDF22-020361-03 Report date: May 05, 2022

Description of tested sample :

Sample Number	SGS sample ID.	Description
1	QDF22-020361.002	Bagged

Physical and chemical testing

Testing results

Testing items	Units	Testing methods	Results	Testing limit of qualification
Proteins	g/100g	GB 4789.3-2016 Method 1	58.1	-
*10-hydroxy-2-decanedioic acid	%	GB 9697-2008 5.3.	3.0	0.1
*Total sugar (calculated based on glucose)	g/100g	Refer to GB 9697-2008	23.5	-
*Acidity	mL/100g	Refer to GB 9697-2008	89.4	-
*Starches	-	Refer to GB 9697-2008	ND	-
*Ashes	g/100g	Refer to GB 9697-2008	1.9	-
*Chloromycetin	Mg/kg	GB/T 18932.19-2003	ND	0.1
Moisture	g/100g	GB 4789.3-2016 Method 2	1.4	-

Notation:

1.ND=Not detected

2.*The testing items are not within the scope of CNAS accreditation that has been approved by our laboratory.

3. The conversion factor for nitrogen to protein is 6.25.

*** End ***

SGS-CSTG Standards & Testing
Services (Qingdao) Co., Ltd.
