# Supplementary material for: Spontaneous pregnancy in a woman with diminished ovarian reserve following dietary supplementation with major royal jelly proteins: A case report
Source: Medicine (Baltimore). 2026 Jun 19;105(25):e49345. doi: 10.1097/MD.0000000000049345 (PMC13286341; doi:10.1097/MD.0000000000049345)
Supplement: Supplementary file 4 [file medi-105-e49345-s004.pdf]

Test Report : QDF22-020361-04

Report date : May 5th, 2022

Description of Tested Sample :

Sample Number	SGS Sample ID	Description
1	QDF22-020361.002	Bagged Sample

Physical and chemical testing

Test results :

Testing items	Units	Testing method	Testing results 002	Limit of quantification
Fusidic acid metabolite(SEM)	µg/kg	GB/T 18932.24-2005	ND	5.0
Nitrofurantoin metabolite (AHD)	µg/kg	GB/T 18932.24-2005	ND	5.0
Furazolidone metabolite (AOZ)	µg/kg	GB/T 18932.24-2005	ND	5.0
Lonydizole.	µg/kg	GB/T 21318-2007	ND	1.0
Metronidazole.	µg/kg	GB/T 21318-2007	ND	0.5
Dimefranidazole.	µg/kg	GB/T 21318-2007	ND	1.0

Notation:

1.ND=Not detected.

END

SGS-CSTG Standards & Testing Services (Qingdao) Co., Ltd.
