# Supplementary material for: Spontaneous pregnancy in a woman with diminished ovarian reserve following dietary supplementation with major royal jelly proteins: A case report
Source: Medicine (Baltimore). 2026 Jun 19;105(25):e49345. doi: 10.1097/MD.0000000000049345 (PMC13286341; doi:10.1097/MD.0000000000049345)
Supplement: Supplementary file 5 [file medi-105-e49345-s005.pdf]

Testing Report QDF22-020361-02 Report date : May 05, 2025

Description of Tested Sample :

Sample Number	SGS Sample ID	Description
1	QDF22-020361.001	Bottled sample

Physical and chemical testing

Testing Results :

Testing items	Units	Testing methods	Testing results 001	Sample limit of quantification
Testosterone	µg/kg	GB/T 21981-2008	ND	20
Methyltestosterone	µg/kg	GB/T 21981-2008	ND	20
Progesterone	µg/kg	GB/T 21981-2008	ND	20
Estriol	µg/kg	GB/T 21981-2008	ND	20
Estrone	µg/kg	GB/T 21981-2008	ND	20
Estradiol	µg/kg	GB/T 21981-2008	ND	20

Notation:
1.ND=Not Detected

*** End ***

SGS-CSTG Standards & Testing
Services (Qingdao) Co., Ltd.
