# Supplementary material for: Spontaneous pregnancy in a woman with diminished ovarian reserve following dietary supplementation with major royal jelly proteins: A case report
Source: Medicine (Baltimore). 2026 Jun 19;105(25):e49345. doi: 10.1097/MD.0000000000049345 (PMC13286341; doi:10.1097/MD.0000000000049345)
Supplement: Supplementary file 7 [file medi-105-e49345-s007.pdf]

检测报告

QDF22-020361-04

发布日期: 2022年05月05日

客户名称: 杭州媛仁生物技术有限公司
客户地址: 杭州市上城区笤帚湾45号7幢114室-5

样品名称: 上媛蜂王浆主蛋白冻干粉
生产商: /
样品批号: /
生产日期: /

以上样品及信息由客户提供及确认, SGS不承担证实客户提供信息的准确性、适当性和(或)完整性责任。

样品接收日期 : 2022年04月18日
检测周期 : 2022年04月18日 - 2022年05月05日
检测要求 : 根据客户要求检测
检测方法 : 请参见下一页
检测结果 : 请参见下一页

除非另有说明，本检测结果仅与被检测物品有关。仅供客户内部使用，不对社会具有证明作用。未经检验机构书面同意，委托人不得擅自使用检测结果进行不当宣传。

扫码查看在线报告
QDF22-020361-04

李敏

SGS授权签字人

通标标准技术服务(青岛)有限公司

Unless otherwise agreed in writing, this document is issued by the Company subject to its General Conditions of Service printed overleaf, available on request or accessible at http://www.sgs.com/en/Terms-and-Conditions.aspx and, for electronic format documents, subject to Terms and Conditions for Electronic Documents at http://www.sgs.com/en/Terms-and-Conditions/Terms-e-Document.aspx. Attention is drawn to the limitation of liability, indemnification and jurisdiction issues defined therein. Any holder of this document is advised that information contained hereon reflects the Company's findings at the time of its intervention only and within the limits of Client's instructions, if any. The Company's sole responsibility is to its Client and this document does not exonerate parties to a transaction from exercising all their rights and obligations under the transaction documents. This document cannot be reproduced except in full, without prior written approval of the Company. Any unauthorized alteration, forgery or falsification of the content or appearance of this document is unlawful and offenders may be prosecuted to the fullest extent of the law.
Attention: To check the authenticity of testing /inspection report & certificate, please contact us at telephone: (86-755) 8307 1443, or email: CN.Doccheck@sgs.com

SGS-CSTC Standards Technical Services (Qingdao) Co., Ltd. SGS Center, No.143, Zhuzhou Road, Laoshan District, Qingdao, Shandong, China 266101 t (86–532) 68999888 www.sgsgroup.com.cn sgs.china@sgs.com

Member of the SGS Group (SGS SA)

检测报告

QDF22-020361-04

发布日期: 2022年05月05日

检测样品描述 :

样品编号	SGS样品ID	描述
1	QDF22-020361.002	袋装样品

理化检测

检测结果 :

检测项目	单位	检测方法	检测结果<br>002	定量限
呋喃西林代谢物 (SEM)	μg/kg	GB/T 18932.24-2005	ND	5.0
呋喃妥因代谢物 (AHD)	μg/kg	GB/T 18932.24-2005	ND	5.0
呋喃唑酮代谢物 (AOZ)	μg/kg	GB/T 18932.24-2005	ND	5.0
洛硝哒唑	μg/kg	GB/T 21318-2007	ND	1.0
甲硝唑	μg/kg	GB/T 21318-2007	ND	0.5
地美硝唑	μg/kg	GB/T 21318-2007	ND	1.0

备注:

1.ND=未检出

*** 结束 ***

Unless otherwise agreed in writing, this document is issued by the Company subject to its General Conditions of Service printed overleaf, available on request or accessible at http://www.sgs.com/en/Terms-and-Conditions.aspx and, for electronic format documents, subject to Terms and Conditions for Electronic Documents at http://www.sgs.com/en/Terms-and-Conditions/Terms-e-Document.aspx. Attention is drawn to the limitation of liability, indemnification and jurisdiction issues defined therein. Any holder of this document is advised that information contained hereon reflects the Company's findings at the time of its intervention only and within the limits of Client's instructions, if any. The Company's sole responsibility is to its Client and this document does not exonerate parties to a transaction from exercising all their rights and obligations under the transaction documents. This document cannot be reproduced except in full, without prior written approval of the Company. Any unauthorized alteration, forgery or falsification of the content or appearance of this document is unlawful and offenders may be prosecuted to the fullest extent of the law.

Attention: To check the authenticity of testing /inspection report & certificate, please contact us at telephone: (86-755) 8307 1443, or email: CN.Doccheck@sgs.com

SGS-CSTC Standards Technical Services (Qingdao) Co., Ltd.

SGS Center, No.143, Zhuzhou Road, Laoshan District, Qingdao, Shandong, China 266101 t (86–532) 68999888 www.sgsgroup.com.cn sgs.china@sgs.com

Member of the SGS Group (SGS SA)
